# Graft versus host disease in humanized mice is differentially controlled by CD28 and CD80/86 antagonists

**DOI:** 10.1186/1479-5876-10-S3-O2

**Published:** 2012-11-28

**Authors:** Nicolas Poirier, Nahzli Dilek, Caroline Mary, Bernard Vanhove

**Affiliations:** 1 Institute of Transplantation Urology Nephrology, University of Nantes, INSERM UMR 643, Nantes, France; 2 Effimune, Nantes, France

## Background

Antagonist anti-CD28 antibodies prevent T-cell costimulation and functionally differentiate from CTLA4Ig since they cannot block CTLA-4 and PDL-1-mediated coinhibitory signals. They demonstrated efficacy in suppressing effector T cells while enhancing regulatory T cells function after organ transplantation.

## Methods and results

Here we evaluated FR104, a novel antagonist pegylated Fab’ anti-CD28 monovalent antibody, in xenogeneic graft-versus-host disease (GVHD) induced in NOD/SCID mice infused with human PBMC and compared efficacy with CTLA4Ig molecules. *In vitro*, FR104 and CTLA4-Ig (LEA29Y) dose-dependently prevented human T cell proliferation to a similar extent in mixed lymphocytes reactions with EC50 at 0.16 and 0.18 micrograms/ml, respectively. IL-2 secretion after stimulation with SEE superantigens was also inhibited in a similar manner.

NOD/SCID mice adoptively transferred with human PBMC presented > 80% engraftment after a week and developed a florid GVHD in the third week due to the proliferation of xenogeneic human T cells and their infiltration into the liver, gut, lung and skin, eventually leading to weight loss and sacrifice. Treatment of recipient mice with 5 mg/Kg FR104 biweekly from day 0 to day 25 reduced target organs infiltration by human T cells and completely prevented weight loss and mortality (p < 0.0001). Mice continued gaining weight and did not succumb even a month after treatment withdrawal. Flow cytometry analysis 2 month post engraftment revealed that FR104 treated mice displayed low level of human cells engraftment (<10%), indicating that targeting the CD28/B7 pathway protected from acute GVHD by inhibiting donor T cell expansion. The therapeutic effect of FR104 was dependent on CTLA-4, since co-administration of FR104 with blocking anti-CTLA4 antibodies completely abrogated FR104-mediated protection. This might be explained by the fact that CTLA-4 signals attenuate T cell responses independently of CD28. In Contrast, biweekly administrations of CTLA4-Ig (LEA29Y or Abatacept), which reduces CD28-CD80/86 costimulation as well as CTLA-4-CD80/86 coinhibition, was ineffective to protect mice from severe GVHD, whereas a less intensive protocol of administration (once a week) was partially effective.

## Conclusion

Our data suggests that antagonist anti-CD28 monovalent antibodies might lead to higher therapeutic indexes, by sparing CTLA-4, as compared to CD80/CD86 antagonists, to dampen T cell activation in transplantation.

